# Serum cross-linked n-telopeptides of type 1 collagen (NTx) in patients with solid tumors

**DOI:** 10.1590/S1516-31802009000100005

**Published:** 2009-05-11

**Authors:** Fernando Jablonka, Fernanda Schindler, Paula Philbert Lajolo, Hélio Pinczowski, Fernando Luiz Affonso Fonseca, Antônio Barbieri, Luiz Henrique Massonetto, Fábio Tadashi Katto, Auro del Giglio

**Affiliations:** 1 BSc. Postgraduate student, Fundação e Faculdade de Medicina do ABC (FMABC), Santo André, São Paulo, Brazil.; 2 MSc. Pharmacist and medical oncologist, Department of Hematology and Oncology, Fundação e Faculdade de Medicina do ABC (FMABC), Santo André, São Paulo, Brazil.; 3 MD, PhD. Medical oncologist, Department of Hematology and Oncology, Fundação e Faculdade de Medicina do ABC (FMABC), Santo André, and postgraduate student, Universidade Federal de São Paulo (Unifesp), São Paulo, Brazil.; 4 MD. Medical oncologist, Department of Hematology and Oncology, Fundação e Faculdade de Medicina do ABC (FMABC), Santo André, São Paulo, Brazil.; 5 PhD. Pharmacist, Fundação e Faculdade de Medicina do ABC (FMABC), Santo André, São Paulo, Brazil.; 6 Nuclear Medicine Physician, Sector of Nuclear Medicine, Hospital Estadual Mário Covas, Santo André, São Paulo, Brazil.; 7 MD, FACP. Chairman of Medical Oncology and Hematology, Discipline of Hematology and Oncology, Fundação e Faculdade de Medicina do ABC (FMABC), Santo André, and Division of Oncology, Hospital Albert Einstein, São Paulo, Brazil.

**Keywords:** Bone neoplasms, Radionuclide imaging, Predictive value of tests, Alkaline phosphatase, Collagen type I., Neoplasias ósseas, Cintilografia, Valor preditivo dos testes, Fosfatase alcalina, Colágeno tipo I.

## Abstract

**CONTEXT AND OBJECTIVE:**

Cross-linked N-telopeptides of type I collagen (NTx) increase in concentration in situations in which bone resorption is increased, such as osteoporosis and bone metastasis (BM). We aimed to evaluate the serum concentrations of NTx in a sample of patients with several types of solid tumors.

**DESIGN AND SETTING:**

Cross-sectional analytical study with a control group in a tertiary public hospital.

**METHODS:**

We performed the quantitative enzyme-linked immunosorbent assay (ELISA) on serum NTx levels in 19 subjects without a history of cancer and 62 patients with various solid tumors who had been referred for a bone scan. Three experienced analysts read all bone scans.

**RESULTS:**

The serum NTx levels in patients with cancer and BM, with cancer but without BM and without cancer were 46.77 ± 2.58, 32.85 ± 2.05 and 22.32 ± 2.90 respectively (P < 0.0001). We did not find any significant correlations of serum NTx with age, gender, history of bone pain, tumor type and bone alkaline phosphatase levels. We found a significant correlation between serum NTx and alkaline phosphatase levels (R^2^ = 0.08; P = 0.022).

**CONCLUSIONS:**

Serum NTx levels are significantly higher in patients with solid tumors and bone metastases than they are in patients without bone metastases and in normal controls.

## RESUMO

**CONTEXTO E OBJETIVO:**

Os N-telopeptídeos do colágeno tipo-I (NTx) elevam-se quando a reabsorção óssea está aumentada, devido a condições como osteoporose e metástase óssea. Sendo assim, temos por objetivo avaliar os níveis séricos de NTx em uma população heterogênea de pacientes com tumores sólidos.

**TIPO DE ESTUDO E LOCAL:**

Estudo transversal analítico com grupo controle, realizado em hospital público terciário.

**MÉTODOS:**

19 pacientes sem história de câncer e 62 pacientes com tumores sólidos de vários tipos, encaminhados para estadiamento e investigação dos sintomas esqueléticos, foram avaliados pela técnica de ELISA (Enzyme Linked Immuno Sorbent Assay) quantitativa para a dosagem de NTx. Três especialistas leram todas as imagens ósseas.

**RESULTADOS:**

O nível de NTx encontrado em pacientes com câncer e metástase óssea, sem metástase óssea e sem diagnóstico de câncer foi 46,77 ± 2,58, 32,85 ± 2,05 e 22,32 ± 2,90, respectivamente (P < 0,0001). Não encontramos correlação entre o NTx, idade, sexo, história de dor óssea, tipo de tumor e níveis de fosfatase alcalina óssea. Encontramos correlação significativa entre os níveis de NTx e de Fosfatase Alcalina (r² = 0,08, P = 0,022).

**CONCLUSÃO:**

O NTx sérico é significativamente mais elevado em pacientes com tumores sólidos e metástases ósseas quando comparado com pacientes sem metástases ósseas e controles normais.

## INTRODUCTION

Bone metastases (BMs) are a frequent event in cancer patients, with a potential for complications such as pain, hypercalcemia and pathological fractures.^1^ The introduction of bisphosphonates in clinical practice has contributed towards decreasing these complications.^2^^,^^3^^,^^4^^,^^5^

Alkaline phosphatase (ALP) has been extensively used to screen patients for BM. However, since ALP can be elevated in other settings, such as liver metastasis, and because it can also be produced by some tumors, high levels of ALP can sometimes be difficult to interpret.^6^ Alkaline phosphatase has several isoforms, including bone alkaline phosphatase (bone ALP), which is produced by osteoblasts and usually contributes about 40% of the total circulating ALP.^6^ In fact, bone ALP is specific for the detection and follow-up of bone diseases that lead to new bone formation, such as BM, in several series in the literature dealing with specific tumor types such as breast,^7^ prostate^8^^,^^9^ and lung carcinomas.^10^

Since BMs induce higher rates of bone resorption, in addition to increased bone formation, evaluation of bone resorption markers in patients with solid tumors who are prone to develop BMs is of interest. Approximately 90% of the organic matrix of bone consists of type 1 collagen, which is a helical protein that is cross-linked at its N-terminal and C-terminal ends. Therefore, the quantitative evaluation of N-terminal cross-linking telopeptides of type 1 collagen (NTx) levels can serve as a marker of increased bone resorption, which may be present in diseases such as osteoporosis and BM.^11^ Urinary NTx levels are indeed increased in patients with solid tumors and BM, and usually decrease after bisphosphonate therapy.^11^^,^^12^ Interestingly, patients in whom urinary NTx levels do not decrease after initial bisphosphonate therapy may be at higher risk of skeletal adverse events such as pathological fractures, and may be candidates for a switch to more potent bisphosphonates.^12^ NTx can also be measured in serum, as a biochemical marker of bone resorption, with similar results to those obtained with urinary NTx.^7^^,^^13^ NTx assays in serum may be more convenient than urine assays, since blood can be drawn simultaneously for all other routine biochemical and hematological tests.

Pectasides et al.^14^ evaluated 33 patients with breast cancer and BM and 31 with metastatic breast cancer and extra-skeletal metastasis and demonstrated that patients with BMs had significantly higher levels of serum NTx than did those without BM. In their study, using a cutoff value of 29.7 nanomoles of bone collagen equivalent per liter (nM BCE) for NTx, the specificity and sensitivity were 87.1% and 45.5%, respectively. In another study, Koizumi et al.^15^ demonstrated that serum NTx was higher in patients with BMs, especially those with extensive skeletal involvement.

## OBJECTIVE

We therefore decided to evaluate serum NTx in an unselected sample of patients with solid tumors who were scheduled to undergo bone scanning, for staging or bone pain evaluation. In so doing, we sought to assess the operating characteristics of serum NTx in a mixed patient population that most closely resembles populations seen at a typical general clinical oncology unit.

## METHODS

After approval by our institutional review board, we prospectively included 62 nonconsecutive patients seen at the Mario Covas Hospital of the Fundação e Faculdade de Medicina do ABC. All these patients granted informed consent for their inclusion in this study.

We included adult patients who had a diagnosis of solid tumors, were not known to have BM and had not yet been subjected to a bone scan. Bone scans were ordered for these patients by the attending physician for the purposes of either staging or evaluation of skeletal complaints.

We excluded patients who had received bisphosphonates, but did not exclude patients who had received previous systemic treatments or radiation therapy. This 62-patient sample had already been used in another study, to evaluate the operating characteristics of serum alkaline phosphatase and bone alkaline phosphatase.^16^

We also included 19 subjects without any history of cancer, recent fractures or osteoporosis. These normal subjects served as controls for serum NTx.

Two to three hours after injection of 1.11 MBq of technetium-99m methylene diphosphonate (^99m^Tc-MDP) at Instituto de Pesquisas Energéticas e Nucleares (IPEN), São Paulo, Brazil, the patients underwent whole body bone scanning. Imaging was performed with a two-headed camera (Forte-ADAC Laboratories, Milpitas, California, United States), equipped with a high-resolution low-energy parallel hole collimator, at 30 cm/min, in a 128 × 128 × 16 matrix size. Images were acquired into a dedicated nuclear medicine computer (Pegasys, ADAC). Energy discrimination was provided by a 20% window centered on the 140 keV photopeak of ^99m^Tc.

Two specialized analysts interpreted each bone scan and a third experienced analyst resolved any discrepancies that arose between the first two readings. The scans were interpreted based on the previous reading experience of these three nuclear medicine physicians as high, intermediate or low probability of BM. Radiographic, tomographic and magnetic resonance imaging examinations were used for additional investigations on suspicious lesions whenever indicated. We conducted a chart review at least six months after the bone scanning procedure, to further subdivide patients into categories of either high or low probability of having BM. We considered that patients had BMs whenever their follow-up showed that there was a fracture, bone pain, a need to start using bisphosphonates or an imaging test that revealed a bone lesion judged to be metastatic.

At the same time as the bone scan, we also measured serum total and ionized calcium, phosphate, magnesium, total protein, albumin, ALP, bone ALP and serum NTx. ALP was measured by quantitative inactivation with *p*-nitrophenyl phosphate substrate (*p*NPP), which yields a normal range for adults from 100 to 290 U/l (Bayer, São Paulo, Brazil). Bone ALP was measured by means of an immunoenzymatic method using the Alkphase-B Kit (Quidel/Metra Biosystems, Mountain View, California, United States). Serum NTx was measured by means of the enzyme-linked immunosorbent assay (ELISA) using the Osteomark NTx^®^ serum test (Whampole Laboratories Inc., Princeton, New Jersey, United States), which is a competitive inhibition assay and was performed as described by Kanakis et al.^7^ The manufacturer’s recommended reference values for women range from 6.2 to 19 nM BCE and, for men, from 5.4 to 24.2 nM BCE.

Associations between categorical variables were tested using the Fisher and chi-squared tests. Associations between continuous variables with normal distribution and between categorical variables were tested using the analysis of variance (ANOVA) test. Regression analysis was used to evaluate correlations between continuous variables. We used the NCCS 2000 statistical package (http://www.ncss.com/) for all statistical calculations.

## RESULTS

We included 62 patients, whose demographic and clinical characteristics are outlined in Table 1. Twenty-seven patients had a high-probability bone scan, but after the chart review conducted six months after the scan, we found that for three of these patients, their physicians did not consider them to have BM. In fact, on later review of these patients’ charts, there was no evidence of any clinical symptoms, skeletal complications, use of bisphosphonates or positive confirmatory imaging tests. We therefore judged that these three patients had not presented bone metastasis. None of the other patients who had either intermediate or low-probability bone scans had any evidence of BM according to any of the above criteria, and they were thus considered not to have BM.

**Figure 1.**
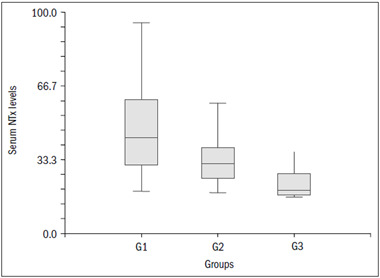
Box plot of serum n-telopeptides of type 1 collagen (NTx) levels in patients with (G1) and without (G2) bone metastasis and in controls (G3).

We also included 19 control subjects (6 males and 13 females), whose mean age was 59.10 ± 3.75 years. The serum NTx levels in patients with cancer and BM, with cancer but without BM and without cancer were (means ± standard deviation): 46.77 ± 2.58, 32.85 ± 2.05 and 22.32 ± 2.90 respectively (P < 0.0001) (Figure 1). When we considered only the patients with cancer, we did not find any significant correlations of serum NTx with age, sex, history of bone pain, tumor type or bone alkaline phosphatase levels. However, we found a significant correlation between serum NTx and ALP levels (R^2^ = 0.08; P = 0.022).

**Table 1.** Clinical and demographic characteristics of the 62 patients included in this study

Characteristic	With bone metastasis	Without bone metastasis	P-value
Patients	24	38	
Age	63.41 ± 2.48	57.44 ± 1.97	0.065
Gender			
Male	9	11	0.48
Female	15	27	
Diagnosis			
Breast carcinoma	12	23	0.55
Prostate carcinoma	15	27	
Others	4	7	
Reason for bone scan			
Pain	7	6	0.20
Staging	17	32	

## DISCUSSION

BMs are a frequent and potentially disabling complication of cancer for which effective preventive measures now exist, such as periodic administration of prophylactic bisphosphonates.^1^^,^^2^^,^^4^ There is therefore a need for early detection of BM, so as to be able to prevent their potentially harmful effects.^3^^,^^5^^,^^17^

Since BMs increase both bone formation and resorption, we decided to test the operating characteristics of serum NTx in a sample of unselected solid tumor patients typical of the type of patient expected to be seen at most general oncology practices. Our results show that serum NTx levels are significantly higher in patients with BMs than in patients with cancer but without BM and in normal controls.

Some authors have shown that high urinary or serum NTx levels should decrease after bisphosphonate treatment, as a sign of the response to antiresorptive treatment.^11^^,^^13^ Therefore, serum NTx evaluation is also potentially useful for following up of patients with BMs who are treated with these antiresorptive agents.

If the serum NTx concentration were to be obtained at presentation from all patients with solid tumors and it were found that the level was normal, it would be most unlikely for such patients to have BMs. Furthermore, if BMs were identified within the context of normal serum NTx concentration, the benefits of bisphosphonates might not be so obvious.^11^ Based on our data, we therefore believe that asymptomatic patients with normal serum NTx levels may be spared from bone scintigraphy.

If, however, the serum NTx levels were abnormal, we would advise patients to undergo bone scintigraphy, especially if simultaneously obtained alkaline phosphatase levels were also abnormal. Such patients should be started on bisphosphonates if one or more BMs are identified. We recommend careful follow-up for patients with high serum NTx levels and a normal scintigram, since such patients may have a higher chance of developing BMs and adverse bone events in the future.^11^^,^^18^ Patients with high serum NTx levels and no BM may also be at risk of losing bone mineral density and should undergo periodic bone densitometry tests, since if osteoporosis is present, it may warrant bisphosphonate treatment even without BM.^19^

## CONCLUSIONS

We conclude that the serum levels of NTx are significantly higher in patients with solid tumors and bone metastases than in those without bone metastasis and in normal controls. Further studies need to be conducted to confirm our findings and evaluate them in relation to new technologies for BM screening, such as fluorodeoxyglucose-positron emission tomography/computed tomography (FDG-PET/CT) scans.
